# fNIRS Outcomes for a Pilot Clinical Trial Combining Frontal tDCS With Walking Rehabilitation in Older Adults

**DOI:** 10.1093/geroni/igaa057.2872

**Published:** 2020-12-16

**Authors:** David Clark, Sudeshna Chatterjee, Jared Skinner, Paige Lysne, Samuel Wu, Ronald Cohen, Dorian Rose, Adam Woods

**Affiliations:** 1 University of Florida, Gainesville, Florida, United States; 2 Appalachian State University, Boone, North Carolina, United States; 3 Aging and Geriatric Research, Gainesville, Florida, United States; 4 University Of Florida, Gainesville, Florida, United States; 5 Clinical and Health Psychology, Gainesville, Florida, United States

## Abstract

This pilot study assessed a novel intervention to enhance both walking and executive function in older adults. The primary hypothesis was that eighteen sessions of frontal lobe tDCS combined with walking rehabilitation would be feasible, safe, and show preliminary efficacy. Eighteen participants were randomized to one of three intervention groups: active tDCS and rehabilitation with complex walking tasks (Active/Complex); sham tDCS and rehabilitation with complex walking tasks (Sham/Complex); or sham tDCS and rehabilitation with typical walking (Sham/Typical). Outcome measures included multiple tests of walking function, executive function, and prefrontal activity during walking as measured by functional near infrared spectroscopy (fNIRS). Of the three groups, the Active/Complex group demonstrated the broadest improvements across outcome measures including for prefrontal activity. The functional range of prefrontal activity in this group was increased considerably, as conceptualized by the Compensation Related Utilization of Neural Circuits Hypothesis. Frontal tDCS is a promising adjuvant to walking rehabilitation.

